# Veterinary Expert Opinion on Potential Drivers and Opportunities for Changing Antimicrobial Usage Practices in Livestock in Denmark, Portugal, and Switzerland

**DOI:** 10.3389/fvets.2018.00029

**Published:** 2018-03-01

**Authors:** Luís P. Carmo, Liza R. Nielsen, Lis Alban, Paulo M. da Costa, Gertraud Schüpbach-Regula, Ioannis Magouras

**Affiliations:** ^1^Vetsuisse Faculty, Veterinary Public Health Institute, University of Bern, Bern, Switzerland; ^2^Department of Veterinary and Animal Sciences, Faculty of Health and Medical Sciences, University of Copenhagen, Copenhagen, Denmark; ^3^Danish Agriculture & Food Council, Copenhagen, Denmark; ^4^ICBAS, Abel Salazar Institute for the Biomedical Sciences, University of Porto, Porto, Portugal; ^5^CIIMAR, Interdisciplinary Center for Marine and Environmental Research, University of Porto, Porto, Portugal

**Keywords:** antimicrobial use, livestock, veterinarians, expert opinion, international comparison, antimicrobial resistance

## Abstract

Reducing antimicrobial use (AMU) in livestock is requested by Public Health authorities. Ideally, this should be achieved without jeopardizing production output or animal health and welfare. Thus, efficient measures must be identified and developed to target drivers of AMU. Veterinarians play a central role in the identification and implementation of such efficient interventions. Sixty-seven veterinarians with expertise in livestock production in Denmark, Portugal, and Switzerland participated in an expert opinion study aimed at investigating experiences and opinions of veterinarians about the driving forces and practices related to AMU in the main livestock sectors (broiler, dairy cattle, fattening/veal calf, and pig industry) of the aforementioned countries. Opinions on potential factors influencing the choice of antimicrobials and opportunities to reduce AMU were collected. Antibiograms are seldom used, mainly due to the time lag between testing and obtaining the results. The perceived percentage of treatment failures varied between countries and livestock sectors; however, little changes were reported over time (2005−2015). The animal health problems of each livestock sector most frequently leading to AMU did not vary substantially between countries. Mandatory official interventions (i.e., binding measures applied by national or international authorities) were highlighted as having the biggest impact on AMU. There was a variation in the experts’ opinion regarding feasibility and impact of interventions both between countries and livestock sectors. Nevertheless, improved biosecurity and education of veterinarians frequently received high scores. Most veterinarians believed that AMU can be reduced. The median potential reduction estimates varied from 1% in Swiss broilers to 50% in Portuguese broilers and veal/fattening calves in all countries. We hypothesize that the differences in views could be related to disease epidemiology, animal husbandry, and socio-economic factors. A profound investigation of these disparities would provide the required knowledge for developing targeted strategies to tackle AMU and consequently resistance development. However, experts also agreed that mandatory official interventions could have the greatest impact on antimicrobial consumption. Furthermore, improvement of biosecurity and education of veterinarians, the use of zinc oxide (in pigs), improving vaccination strategies, and the creation of treatment plans were the measures considered to have the largest potential to reduce AMU. This paper can inform policymakers in Europe and countries with a similar animal production regarding their AMU policy.

## Introduction

Reducing antimicrobial use (AMU) in animal production is currently a priority within the Veterinary Public Health sphere. The potential risks for human health associated with AMU in animals have urged European institutions to consider it a critical issue to be addressed in the near future (1–3). On a global scale, veterinary antimicrobial consumption estimates for the future are not optimistic (4). Furthermore, the importance of antimicrobial resistance was underpinned by the recent United Nations high level meeting on antimicrobial resistance. This was only the fourth time in history that a health issue was brought up to the United Nations General Assembly (5).

Despite the fact that the actual public and animal health burdens related to AMU in animals remain unknown (6–8), it is generally accepted that a more prudent use of these substances should be achieved in the veterinary field. Nonetheless, interventions to reduce AMU need to be based on scientific evidence and to be feasible and efficient, with the minimum possible impact on production. Diseased animals require handling and antimicrobials often form part of an effective treatment, and therefore inappropriate reductions in AMU might raise animal welfare issues.

Overall, a reduction on antimicrobial sales has been observed in Europe over the past few years (9), with success stories occurring in several countries (10–14). A thorough understanding of what drives AMU needs to be obtained—from the reasons that lead to the need of antimicrobials to the most impactful and feasible interventions to reduce AMU. The overall levels of antimicrobial sales are clearly different across Europe (9). Taking the countries participating in this study as an example, in 2014 veterinary antimicrobial sales in Denmark totalized 44.2 mg per population correction unit (mg/PCU); in Switzerland the sales value was of 56.9 mg/PCU, while in Portugal antimicrobial sales reached 201.6 mg/PCU (9). Furthermore, sales of different antimicrobial classes and product pharmaceutical formulations are also highly variable between countries (9, 15). It is therefore likely that a multitude of factors shape prescription practices. These are probably related, among other reasons, to country idiosyncrasies, presence of infection and management practices, or characteristics of the different livestock production sectors. A comprehensive understanding of these factors is of paramount importance to tackle antimicrobial consumption, if cost-effective measures are to be identified or developed.

Veterinarians are key stakeholders in the AMU topic. Besides being authorized to prescribe antimicrobials, veterinarians also advise farmers on animal health and production management issues that can strongly influence the need for antimicrobial treatment at farm level. In addition, veterinarians have a central position—as a communication bridge—between farmers and authorities. The “on-farm” knowledge veterinary practitioners possess is a precious resource of great relevance for elucidating policymakers on the best strategies to optimize AMU.

Expert opinion represents a scientific method to collect data and inform decision makers (16). This method can provide insightful information of great relevance. The objective of this study was to explore the experiences and opinions of veterinary experts about factors related to AMU and the opportunities to minimize the use of these compounds in livestock production. Moreover, we aimed to compare our findings between different European countries and livestock sectors. This might also help to identify drivers of AMU across Europe and specific areas to be targeted by interventions. Furthermore, successful strategies identified in one country could serve as a paradigm to others aiming to design future interventions.

## Materials and Methods

An expert opinion study was conducted in three European countries: Denmark, Portugal, and Switzerland, which represent different levels of antimicrobial sales (Portugal—above European average; Switzerland and Denmark—below European average) (9) and distinct geographical locations (Northern Europe, Central Europe, and Southern Europe). We included veterinarians from four different livestock sectors: broiler, swine, dairy cattle, and veal/fattening calf production. In Switzerland, the veal calf (slaughtered around 6 months of age) sector was targeted instead of the fattening calf (slaughtered around 10–12 months of age) production system due to its greater relative importance compared with other countries, and the higher level of antimicrobial consumption observed in this age group of cattle (17).

### Selection of Participants

Veterinary experts on AMU and animal production were identified by contacting academic departments related to animal production and clinics, veterinarians, and farmers’ associations. As commonly used in expert elicitation studies, the snowball effect was applied: initially selected experts were asked to provide suggestions of potential participants (16). We aimed at having five to nine experts per stratum as suggested in the literature (16). Potential participants were contacted and if agreed to take part, further details about the study were provided and the questionnaires were sent. A total of 67 veterinarians by training were enrolled as experts (Denmark: *n* = 18, Portugal: *n* = 25, and Switzerland: *n* = 24).

### Questionnaires

Four different questionnaires were developed in MS Excel (18), one for each targeted livestock sector. The questionnaires were written in English; however, experts could provide comments or answers in their native language in the case of open questions. The objectives of the study were communicated to all participants by electronic mail. Participants were also informed that their data would be used for a scientific publication. All experts were assured anonymity. In accordance with the institutional requirements and local legislation, no ethical approval was necessary.

The structure of the questionnaires followed a given order:

(1) Personal data: information on the participants’ professional activity;
(2) Factors related to antimicrobial prescription: the questions addressed potential influencing factors on prescription practices, such as the use of antimicrobial susceptibility testing (AST), treatment failures (defined as an antimicrobial treatment that did not work—a treatment that did not cure the animal from a given condition), and animal health;
(3) Opportunities to change: the questions aimed to obtain data on the veterinarian’s perspective on the best actions to reduce AMU at the farm level. The list of interventions can be consulted in Table 1.

**Table 1:** List of interventions aiming at the reduction of antimicrobial use (AMU).

Abbreviation	Intervention
ban	Ban the veterinary use of more antimicrobials substances/classes
benchf	Benchmarking strategies on antimicrobial use at the farm level with penalties above a certain limit
benchv	Benchmarking strategies on antimicrobial use for veterinarians with penalties above a certain limit
dens	Operate with an optimal number of animals per farm
dxpath	Improve the diagnostic methods for pathogens (cost, timeliness, sensitivity, specificity)
econ	Reduce economic support to farms with higher antimicrobial usage
eduf	Improve farmer’s education
eduv	Improve veterinarian’s education on the topic
extbio	Improve farm external biosecurity
feed	Improve feed quality
guide	Creation of prescription guidelines/protocols for veterinarians
illegal	Control of illegal trade, of the amounts imported and sales/offers of antimicrobials directly to the farmer
intbio	Improve farm internal biosecurity/hygiene
label	Labeling strategies (e.g., labels for products from animals raised organically)
probio	Modify animals’ intestinal flora through the use of probiotics/prebiotics
profit	Limit veterinarian’s profit from antimicrobial sales
st	Improve antimicrobial susceptibility testing (cost, timeliness, sensitivity, specificity)
trade	Sales/trade restrictions for farms with high antimicrobial usage
txplan	Creation of farm treatment plans
vac	Improve farm vaccination strategies
water	Improve water quality
zinc	Use of zinc

*Experts assessed the impact on AMU and the feasibility of the interventions included in this list. The same abbreviations as above are used in Table 4 and Datasheet S3 in Supplementary Material*.

Factors (second group of questions) and interventions (third group of questions) were discussed and defined by the authors, based on their experience and the existing literature. Participating veterinarians were asked to answer the questions based on their knowledge on the livestock sector in their respective countries and not solely based on their clinical experience. The questionnaires are available in Datasheet S1 in Supplementary Material.

The questionnaires were pre-tested with one expert from each of the four livestock sectors in September 2015. Because no influential changes were made to the questionnaires following pre-testing, three completed pre-test questionnaires were included in the analysis. The fourth expert who was selected for the pre-testing procedure provided oral feedback on the content of the questionnaire without completing it; therefore, this questionnaire was not included in the analysis. The questionnaires were sent via electronic mail and reminders followed 1 and 2 months after the expert agreed on participating. Data collection took place between October 2015 and March 2016. Experts were offered support if doubts related to the questionnaire arose during its completion. Participants were rewarded with a bottle of wine (Portugal and Switzerland) or a gift card (Denmark).

### Analysis

Preliminary data management was done in MS Excel (18). Data analysis was performed using the statistical software R version 3.3.1 (19). Results were stratified per country, per livestock sector and per country and livestock sector combined and summarized using the mean, SD, median, and 25−75% interquartile range (IQR).

## Results

### Personal Data of Experts

Overall, the median number of years of clinical experience was 17 (IQR: 10−25). The percentage of participants working at university hospitals was 19%, with differences existing between countries and livestock sectors (Datasheet S2 in Supplementary Material).

From the 67 participants, 6 were currently not practicing clinical veterinary medicine (4 Danish veterinarians—1 pig, 1 veal, and 2 dairy veterinarians—and 2 Portuguese veterinarians—1 broiler and 1 pig veterinarian). However, all participants’ answers were included given their vast expertise on the topic and past long-term clinical veterinary experience. Furthermore, their professional areas of activity (industry and disease prevention consultancy) allowed them to work closely with farmers and practitioners and being up to date about the countries’ situation with respect to antimicrobial consumption in livestock.

### Factors Related to Antimicrobial Prescription

#### Antimicrobial Susceptibility Testing

**Figure 1:**
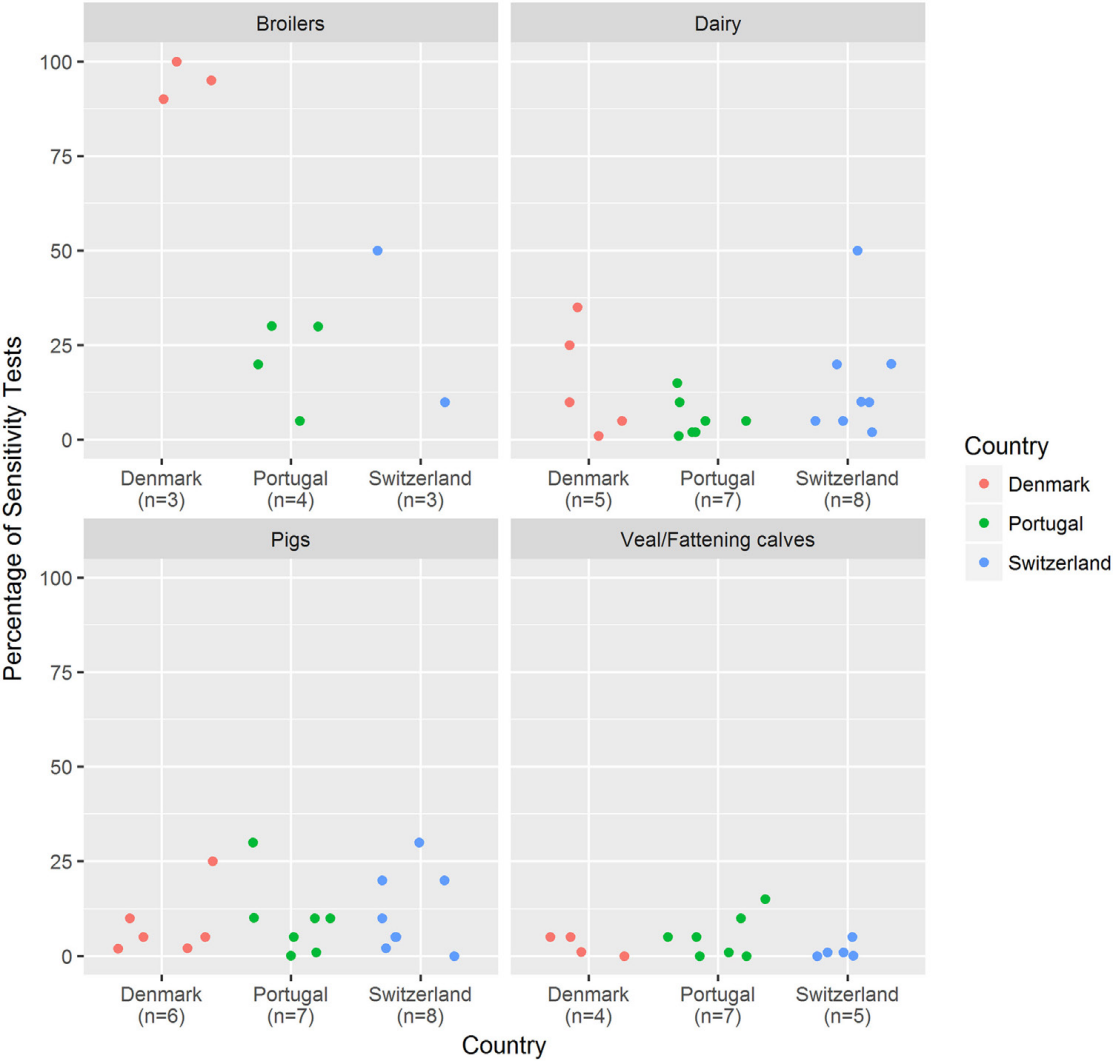
Proportion of antimicrobial treatments based on a previous sensitivity testing. Veterinary experts (*n* = 67) were asked to indicate the proportion of antimicrobial treatments for which an initial sensitivity test was performed in advance. Results are stratified per country and livestock sector. *n*, number of answers.

The percentage of AST before prescribing antimicrobials did not vary greatly between countries, with an overall median value of 5% for every country. Broiler experts appeared to use AST more often than veterinarians working with the other species (Figure 1).

Experts were asked to score potential reasons for not conducting AST more often, on a scale from 1 (low importance) to 10 (high importance). There were no clear differences between the factors that experts considered more relevant, neither at the country nor at sector level. The “time lag between sampling and obtaining the result” and the fact that “AST results do not help on the clinical decision making process (or on the selection of the antimicrobial to be used)” were the factors with the highest overall median score (9 and 8, respectively) (Figure S1 in Supplementary Material).

#### Treatment Failures

In most livestock sectors (all except dairy cattle, for which Swiss experts described a higher median), Portuguese experts reported the highest proportion of treatment failures (Table 2). With the exception of Switzerland, fattening calves were the production sector where treatment failures were reported most frequently.

**Table 2:** Median and 25–75% interquartile range of reported antimicrobial treatment failures for 2015.

Country	Species	Median (%)	25–75% quartiles (%)	*n*
Denmark	Broilers	10	NA	3
	Pigs	5	3−9	6
	Dairy	2	NA	3
	Fattening calves	15	NA	4

Portugal	Broilers	70	–NA	3
	Pigs	20	13−30	7
	Dairy	15	13−20	7
	Fattening calves	25	20−31	7

Switzerland	Broilers	2	NA	3
	Pigs	10	6−10	7
	Dairy	20	12−30	8
	Veal calves	10	10−15	5


*Not all 67 participating veterinarians answered for the 3 years. 25–75% quartiles were not computed for sample sizes lower than 5*.

*n, number of answers*.

To verify if the experts had perceived an increase or decrease of treatment failures throughout time, participants were also asked about the proportion of treatment failures in 2005 and 2010. Around half (52%, 32/62) of the respondents presented the same percentage of treatment failures for the different years, whereas 34% (21/62) reported an increase. This value was particularly high in Portugal (52%, 13/21). For 80% (12/15) of the Danish respondents, the percentage of treatment failures remained the same during the 10-year period.

Experts were asked to name up to three antimicrobial classes that most frequently lead to treatment failures (Figure S2 in Supplementary Material), as well as up to three classes for which there was a larger increase in treatment failures from 2005 to 2015 (Figure S3 in Supplementary Material). Overall, no clear patterns were detected, however, some similarities between countries were observed for the classes most frequently leading to treatment failures in dairy cattle.

#### Animal Health

Experts were asked to name up to five diseases that most frequently lead to the use of antimicrobials. The experts had the chance to specify the age period, but this variable was removed from the analysis to reduce the number of strata. A total of 309 answers was obtained. Twenty-three replies were considered missing values (e.g., the expert did not complete the five options) and three were excluded for referring to poor management practices (as no link to potential diseases or syndromes could be established). If respondents were not able to mention a specific disease or pathogen (e.g., colibacillosis), they had the choice to indicate clinical signs or syndromes (e.g., diarrhea, pneumonia) instead. Overall, 38% (118/309) of the answers included specific diseases or pathogens (Table 3).

**Table 3:** Proportions of specific diagnoses associated with the application of antimicrobials per country/livestock sector.

	Broilers		Dairy cattle		Pigs		Veal/Fattening calves		Total	
	Specific (%)	*n*/Total	Specific (%)	*n*/Total	Specific (%)	*n*/Total	Specific (%)	*n*/Total	Specific (%)	*n*/Total
Denmark	71	10/14	24	6/25	57	17/30	26	5/19	43	38/88
Portugal	50	10/20	6	2/25	71	25/35	31	10/32	39	47/122
Switzerland	85	11/13	16	6/38	47	15/32	5	1/19	33	33/99
Total	66	31/47	14	14/98	59	57/97	23	16/70	38	118/309

*Each of the 67 participating veterinarians could mention up to five diseases/pathogens (specific diagnosis) or clinical signs/syndromes (unspecific diagnosis). The results represent the proportion of specific diagnoses mentioned by the participants*.

*n, number of answers*.

**Figure 2:**
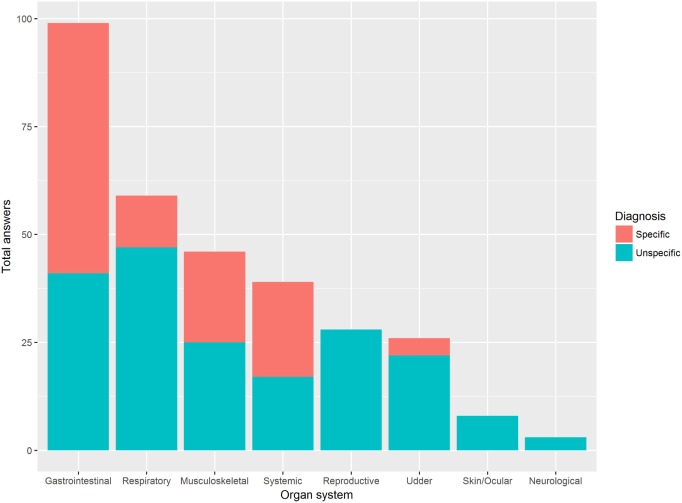
Proportion of specific and unspecific diagnosis per organ system/syndrome. Veterinary experts (*n* = 67) were asked to list the five main diseases that lead most often to the use of antimicrobials. If they were not able to mention a specific pathogen—specific diagnosis—they could refer to a syndrome instead (e.g., diarrhea, pneumonia)—unspecific diagnosis. Results are presented irrespectively of the country/livestock sector and grouped per organ system.

Respondents’ answers were grouped into the following categories: gastrointestinal, musculoskeletal, neurological, reproductive, respiratory, skin/ocular, udder, systemic, and prophylactic treatments where antimicrobials were used. Gastrointestinal diseases were the ones indicated most often and in 41% (41/99) of the times the experts did not mention a specific disease (Figure 2).

**Figure 3:**
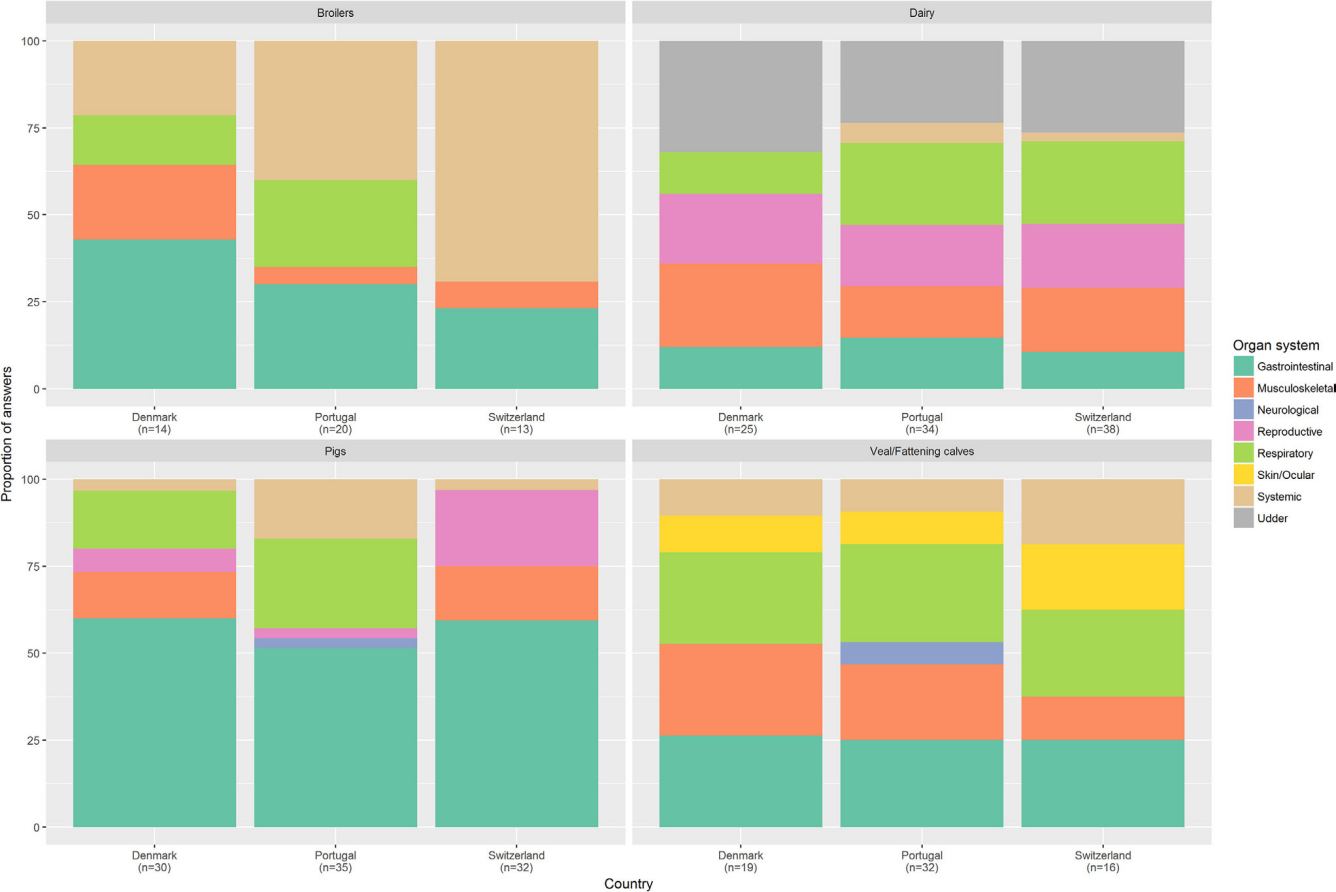
Diseases or syndromes that most frequently lead to the use of antimicrobials, grouped by organ systems. Veterinary experts (*n* = 67) were asked to name up to five main diseases (or syndromes in case they could not mention a specific pathogen) that most frequently lead to the use of antimicrobials. Results were grouped per organ system and are presented for each country and livestock sector. *n*, number of answers.

For broilers, Danish experts tended to report gastrointestinal diseases as the leading cause for the use of antimicrobials, while in Portugal and Switzerland systemic problems, mostly yolk sac infections and colisepticaemia, were the most frequently reported health issues. Furthermore, all Portuguese veterinarians mentioned broiler respiratory problems as one of the five main diseases. No Swiss and only one Danish broiler expert mentioned respiratory diseases (Figure 3).

Dairy experts agreed that the diseases most frequently leading to the use of antimicrobials were either related to the udder (*n* = 16) or to the respiratory tract (*n* = 4) (Figure 3). Mastitis or drying-off were reported as the reasons for treatment that most frequently led to the use of antimicrobials by 16 of 21 experts.

For veal/fattening calves: 100% of Danish (4/4), 60% of Swiss (3/5), and 57% (4/7) of Portuguese experts referred to respiratory diseases as the main cause for AMU.

Regarding pigs, systemic and respiratory problems had a higher importance in Portugal than Denmark or Switzerland (Figure 3). *Lawsonia* spp. infection was mentioned more frequently by Danish experts (9/30 answers) than in Portugal (3/35) or Switzerland (3/40).

### Improving the Use of Antimicrobials

#### Effect of Interventions Applied by Different Stakeholders

**Figure 4:**
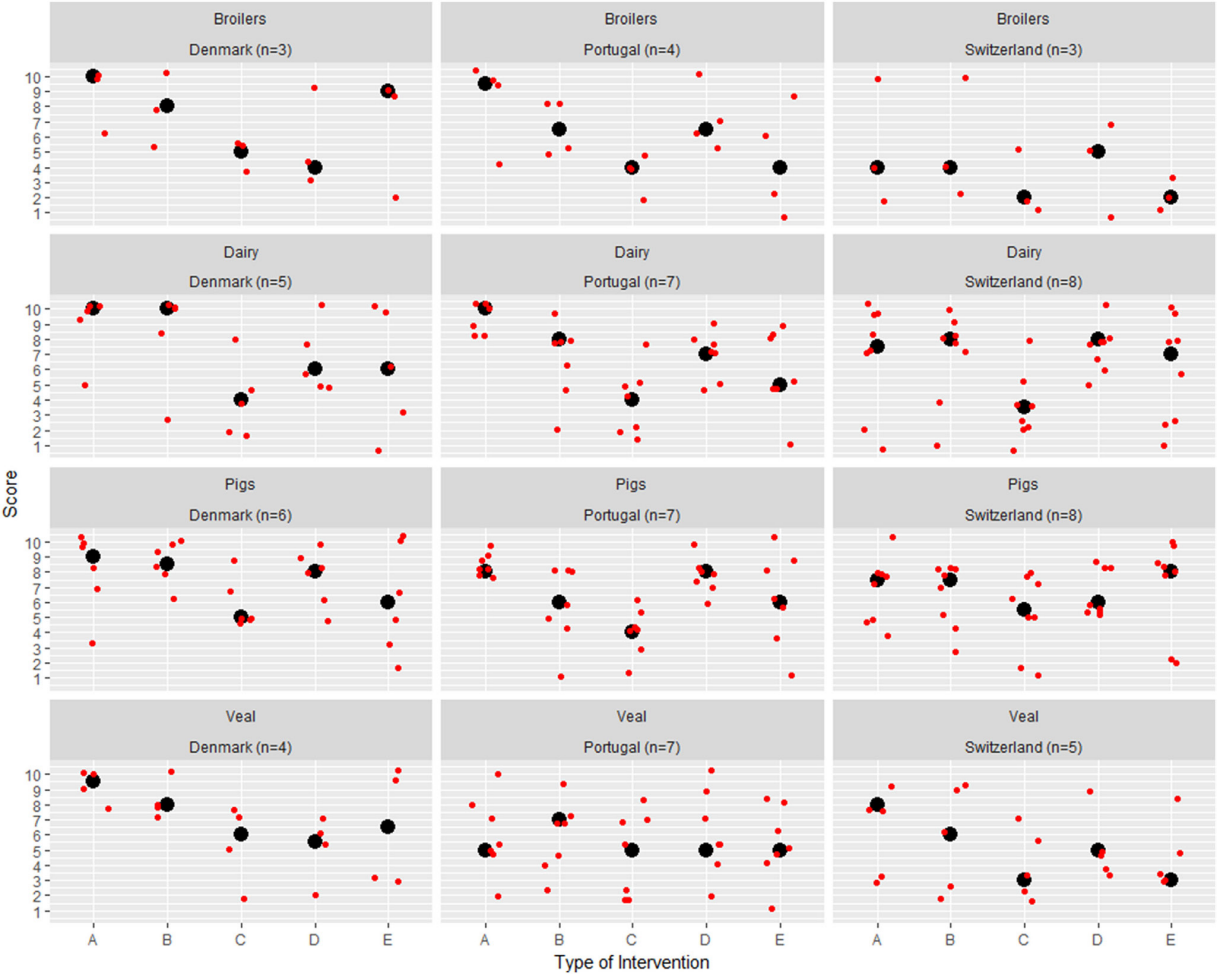
Types of intervention that have a higher potential to reduce antimicrobial use (AMU). Veterinary experts (*n* = 67) were asked to score from 1 to 10 (1—low importance; 10—high importance) the potential impact of five types of interventions to reduce AMU. Red dots represent the individual answers of each expert. Black dots represent the median values. **(A)** “Interventions from European/National Authorities (mandatory application)”; **(B)** “Interventions from the agricultural associations (mandatory application)”; **(C)** “Interventions from the agricultural associations (voluntary application)”; **(D)** “Interventions suggested by the veterinarian”; **(E)** “Intervention made by the farmer on an individual basis.” *n*, number of answers.

According to the consulted experts, mandatory interventions (i.e., binding measures applied by national or international authorities) appeared to work best in reducing antimicrobial consumption, on both country- and species-level. No large differences were observed between the intervention preferences for different livestock sectors (Figure 4). “Interventions from the farm associations (voluntary application)” were scored slightly higher in Denmark. It was also possible to note a high variability on the “interventions made by the farmer on an individual basis” (i.e., measures introduced by the farmer at his/her own initiative to reduce AMU at the farm level, such as additional disease testing, quarantine, reducing stock density, or improving housing conditions) across all countries.

#### Interventions to Reduce AMU: Impact and Feasibility

Participating veterinarians were asked to score from 1 (lowest) to 10 (highest) the feasibility and impact of 21 (for broilers, dairy cattle, and veal/fattening calves) or 22 (for pigs—use of zinc oxide was added as an additional option) potential measures aiming at reducing AMU at the farm level. Feasibility and impact values were averaged creating a combined score. Both for feasibility and impact, as well as for the combined score, differences were found at country and species level (Datasheet S3 in Supplementary Material). Overall, “improve farm internal biosecurity/hygiene,” “improve veterinarians’ education on the topic,” and the “use of zinc” (for pigs) were the measures with the highest combined mean score of feasibility and impact (Table 4). On the other hand, the following interventions scored the lowest: “control of illegal trade, of the amounts imported and sales/offers of antimicrobials directly to the farmer,” to “modify animals’ intestinal flora through the use of probiotics/prebiotics,” and to “limit veterinarians’ profit from antimicrobial sales.” However, it should be stressed that exceptions existed for some country/livestock sector combinations (Datasheet S3 in Supplementary Material).

**Table 4:** Impact and feasibility of interventions to reduce antimicrobial use considering all the veterinary sectors and countries together.

	Score			Feasibility			Impact			
Variable	Rank	Mean	SD	Rank	Mean	SD	Rank	Mean	SD	*n*
intbio	1	7.6	1.4	6	6.6	2.1	1	8.5	1.6	66
eduv	2	7.3	1.4	4	7.2	2.0	4	7.3	2.0	66
zinc	3	7.2	2.4	3	7.2	3.4	7	7.1	2.6	21
vac	4	7.1	2.0	2	7.3	2.2	10	6.9	2.5	66
txplan	5	7.1	1.8	1	7.3	2.2	13	6.8	2.4	66
eduf	6	6.9	1.6	9	5.9	2.2	2	7.9	1.7	66
extbio	7	6.4	1.7	14	5.6	2.2	6	7.2	2.5	64
feed	8	6.3	1.7	13	5.7	2.2	11	6.9	2.3	66
benchf	9	6.2	2.2	15	5.6	2.7	12	6.8	2.6	66
guide	10	6.2	2.0	5	6.7	2.6	18	5.7	2.5	66
water	10	6.2	2.2	7	6.3	2.4	16	6.0	2.9	66
dxpath	12	6.1	1.8	16	5.2	2.2	8	7.1	2.4	66
trade	13	6.1	2.1	19	4.9	2.6	5	7.3	2.3	66
econ	14	6.1	2.0	17	5.2	2.7	9	7.0	2.5	66
dens	15	6.0	1.8	21	4.7	2.4	3	7.4	2.2	66
ban	16	5.7	2.1	8	6.0	3.0	19	5.6	3.0	65
st	17	5.6	1.7	18	5.0	2.2	15	6.2	2.4	66
benchv	18	5.5	2.2	20	4.9	2.6	14	6.2	2.7	66
label	19	5.5	2.0	11	5.8	2.6	20	5.2	2.7	66
profit	20	5.3	2.8	10	5.9	3.4	21	4.7	3.4	65
probio	21	5.2	2.3	12	5.7	2.9	22	4.6	2.4	66
illegal	22	4.9	2.2	22	3.9	2.7	17	5.9	3.2	66

*n, number of experts providing an answer. The variables are identified by an abbreviation (please see Table 1 for further information)*.

#### Potential for Reduction

**Figure 5:**
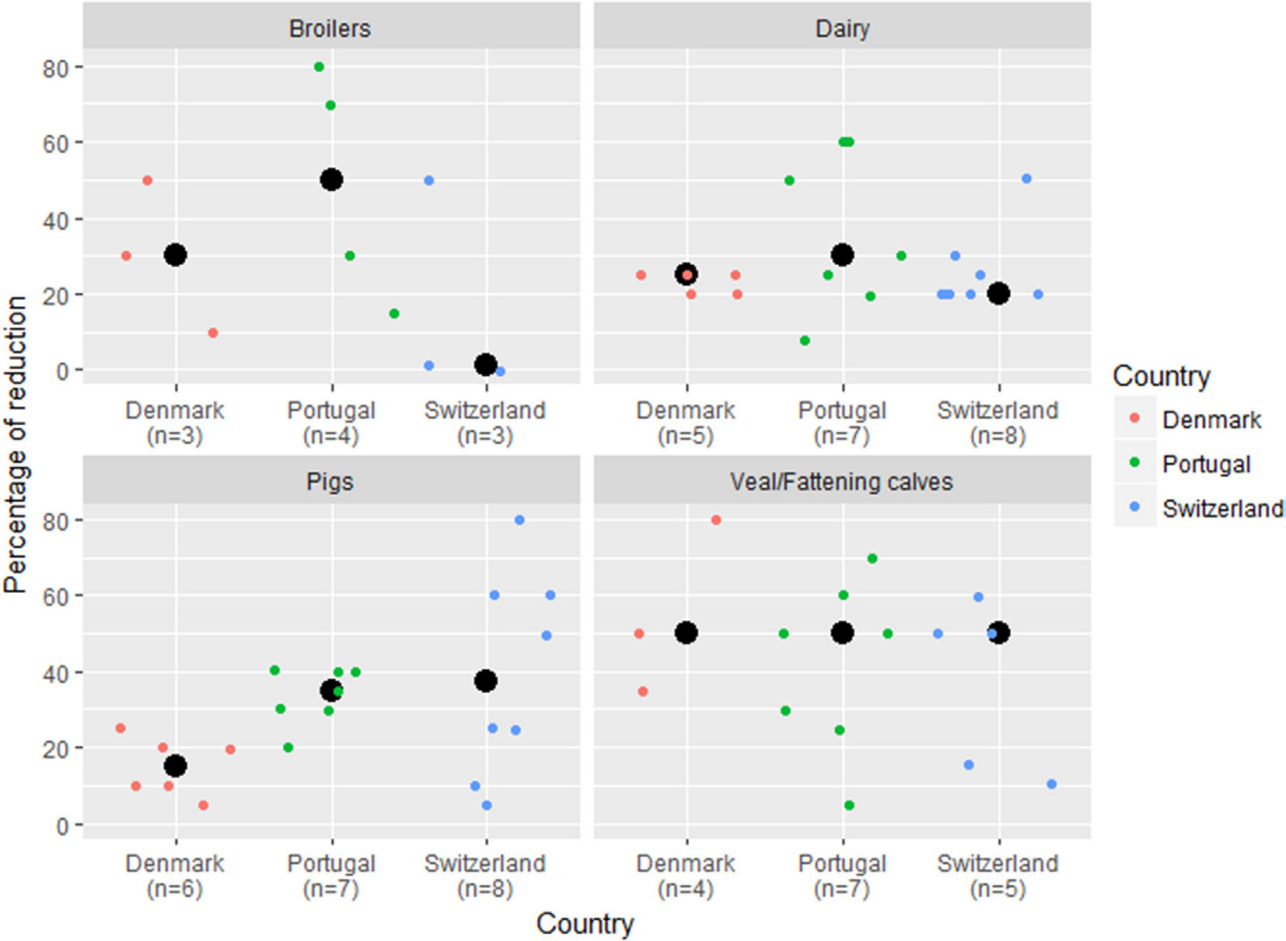
Percentage of reduction of antimicrobial use that the veterinary experts believe can be achieved in their country/livestock sector. Veterinary experts (*n* = 67) were asked which would be the potential reduction of antimicrobial consumption in their country/livestock sector. Results are stratified per country and livestock sector. Colored dots represent individual answers. Black dots represent the median values. *n*, number of answers.

Veal/fattening calf experts suggested the largest (median of 50%) potential for reduction in AMU (Figure 5). The estimates from broiler veterinarians were the ones varying the most between countries, from a median of 1% in Switzerland to 30% in Denmark, and 50% in Portugal. In the pig sector, Danish experts suggested a median cut of 15%, compared with 35% in Portugal, and 38% in Switzerland. In the dairy sector, values ranged from 20% in Switzerland to 25% in Denmark, and 30% in Portugal. One Swiss broiler expert mentioned that the potential to reduce AMU was null. Danish experts provided estimates with lower variability when compared with their colleagues from Portugal and Switzerland (Figure 5).

## Discussion

We found differences in the veterinarians’ opinions and perceptions of the use of antimicrobials both at the country and at the livestock sector level. Some of the observed discrepancies can be explained by the characteristics of each livestock sector, while others could potentially reflect differences between countries in the drivers and attitudes toward the use of antimicrobials and therefore require further investigation.

### Factors Related to Antimicrobial Prescription

Overall, AST was rarely used before prescribing antimicrobials (Figure 1). This is in accordance with results from other studies reporting low use of AST in veterinary practice (20, 21). Nevertheless, the percentage of prescriptions based on prior antibiograms was higher for broilers than for other species. We believe that this relates to the characteristics of poultry production, where the application of an ineffective treatment to the entire flock might jeopardize a high number of animals and cause large economic losses. In addition, when parental stocks are diseased, this is usually followed by the identification of the causative pathogen together with its resistance profile. If the same problem occurs in broilers from that lineage, the most effective treatment can therefore be applied immediately. It should also be stressed that pathological investigations are more effortlessly conducted in poultry production than in other livestock industries and, thus, it might be easier to collect samples for AST. A potential measure, which could be recommended to increase the use of AST, would be to allow the administration of certain antimicrobial classes only in combination with a prior antibiogram. The same requirement could be implemented, when providing a treatment other than that suggested by official treatment guidelines. Such a measure is already in place in several countries; in Denmark, for instance, mastitis treatments with antimicrobials other than penicillins have to be preceded by AST (22).

Waiting time for AST results was reported to discourage the use of these tests. Other studies have already highlighted the importance of having rapid AST (20, 21, 23). The pressure exerted by farmers toward antimicrobial prescription, as well as the economic costs are often addressed in studies that investigate the prescribing behavior of veterinarians (20, 21, 23–26). In our study, both factors did not score as high as the “time lag between sampling and obtaining the result.” Another aspect to consider is the reliability of the method, as well as the relevance of *in vitro* tests in a clinical setting (21). There is a need to adapt AST to/toward the veterinary clinical context (e.g., developing the guidelines for interpretation of AST test results, especially on veterinary pathogens). Furthermore, when the test is applied, there is no guarantee that the susceptibility profile reported originates from the pathogen causing disease. This could result from a contaminated sample or by culturing an agent that is not the cause of the disease in question, such as in coinfections.

Treatment failures constitute a potential economic loss for producers (27). Taking action to reduce the occurrence of treatment failures might be an intervention of interest for both, public health authorities (potential for reducing AMU and therefore the selection and emergence of resistant bacteria), as well as producers (potential for reducing treatment costs and production losses related to prolonged disease morbidity). At country-level, the percentage of treatment failures reflected the resistance situation in zoonotic and indicator bacteria in this study. Portuguese experts reported the highest level of failures and, in general, Portugal presents the highest prevalence of resistant bacteria. In Denmark, where the prevalence of resistance is generally the lowest among the three countries, veterinarians perceived a lower level of treatment failures (28–37). We cannot exclude the possibility that the experts were biased by their knowledge on the country situation in terms of antimicrobial resistance. It should also be stressed that antimicrobial resistance is only one of many factors that potentially contributes to treatment failures. Other reasons, such as incorrect diagnosis, wrong choice of antimicrobial, wrong choice of dosage, incorrect application, or timeliness of treatment, also play a role and could influence the results we obtained.

There were some similarities between the antimicrobial classes that experts highlighted as the ones leading most frequently to treatment failures and the relative consumption of antimicrobials for pigs in Denmark and Switzerland: penicillins were more frequently mentioned by Danish experts than Swiss, probably reflecting that penicillin has a high relative consumption in Denmark (15); while sulfonamides and trimethroprim have a higher consumption in Swiss pigs and were also named more frequently by Swiss experts as contributing to treatment failures. Such a pattern was not so evident for dairy and veal/fattening calves, but it should be noted that antimicrobial consumption estimates were available for the entire cattle population and not just for the individual production sectors (15). Nevertheless, it should be noted that for assessing the link between AMU and the antimicrobial classes highlighted by the experts, treatment incidence metrics would be more appropriate than the relative consumption of antimicrobial classes. Unfortunately, such data are yet not available at national level in Switzerland. Portugal could not be included in this comparison given that no consumption estimates at the species level were available.

Animal health is a key factor to consider when investigating the differences in AMU patterns across different countries. A large proportion of answers included symptoms and non-specific diseases/infections. This might indicate that veterinarians sometimes prescribe antimicrobials without confirming the pathogen causing the observed disease or due to coinfections, where several pathogens may be involved. Treating without knowing the exact agent could reflect the pressure to contain a disease rapidly to avoid its spread within the herd. Additionally, Coyne et al. reported that UK pig surgeons use antimicrobials not only to cure but also to prevent disease (23). Furthermore, several studies highlight experience as an important factor influencing the veterinarian’s prescription behavior (20, 21, 23). No large differences were noted on the organ systems that most frequently require the use of antimicrobials, especially for dairy and fattening/veal calves. An example of expert agreement is reflected on the emphasis given to udder treatments. This is in line with the results obtained by De Briyne et al., which stated that mastitis was the most frequent indication for antimicrobial prescription in dairy cattle (38). Measures to improve udder health (e.g., improved milking hygiene) could therefore result in a drop of AMU in dairy cattle.

Some differences were detected with respect to the specific animal health problems leading to the use of antimicrobials. For pigs, it is interesting to note that Danish experts referred to *Lawsonia* spp. more often than other countries’ experts. We hypothesize that this could potentially be related to: (a) misdiagnosis by the veterinarians (e.g., identifying *Lawsonia* spp. in diarrhea cases when *Lawsonia* spp. is present, but not necessarily the causative agent); (b) more attention to the negative impact of *Lawsonia* spp. on production; (c) an antimicrobial consumption pattern in Portugal and Switzerland that could eventually mask the presence of these bacteria (e.g., use of pleuromutilins against swine dysentery will combat *Lawsonia* spp. too) (39); or (d) a higher occurrence of clinical cases of *Lawsonia* spp. in Denmark. For broilers, respiratory problems were reported with a higher frequency in Portugal than in the other countries. Besides a potentially higher prevalence of infectious diseases, respiratory problems in Portuguese broiler flocks might relate to management practices, in particular deficient ventilation and heating management. Suboptimal climate conditions have been previously identified by livestock veterinarians as a catalyzer of AMU (40).

### Opportunities to Improve

From the veterinarians’ perspective, official mandatory interventions (from national or international authorities) were the ones with the largest potential for reducing AMU. Nonetheless, it is interesting to note that national plans might not always be well-received by veterinarians initially, as noted by Postma et al. with regard to Flemish veterinarians (40). “Interventions from the farm associations (voluntary application)” received a higher score in Denmark than in Portugal or Switzerland (Figure 4). This might reflect the Danish veterinarians’ experience on past successful interventions driven by the industry, such as the voluntary ban on third and fourth generation cephalosporins in pigs (41). It is also interesting to note that “interventions made by the farmer on an individual basis” or “interventions suggested by the farm veterinarian” were perceived to have a limited potential to reduce AMU. We hypothesize this is related to potential differences on farmers’ motivation to change, as experienced by the experts. Therefore, mandatory interventions (e.g., benchmarking systems such as the Danish “Yellow card” scheme, or binding legislation aiming at improving biosecurity) might have to be included when developing national plans to reduce AMU. The role of targeted vaccination should also be further explored. Other actions may involve eradication of infections—such as dysentery in pigs or bovine viral diarrhea in cattle; and here a collaboration between the veterinary authorities and the livestock industry may be required. Finally, we recommend that some degree of check of compliance with the legislation/sector requirements put in place to lower AMU should be undertaken—either by the authorities or through third party independent auditors. This is already in place in Denmark.

With respect to the score of interventions to reduce consumption, some common features were observed in the answers from the respondents. Biosecurity measures (especially internal biosecurity) and improving veterinarians’ education on this topic, received high scores rather consistently throughout different countries and livestock sectors (Datasheet S3 in Supplementary Material). The latter reflects the importance of raising awareness in all the stakeholder groups. Programs to increased awareness about AMU and antimicrobial resistance were hypothesized as a contributing factor to the reduction of antimicrobial sales in Switzerland over the last years (12). A veterinary education (during graduation and in continuous education programs) that stresses the importance of prudent AMU is expected to have positive effects on the veterinary practices. The focus on biosecurity highlights the importance of preventing disease, rather than curing it, hence reducing the need for the use of antimicrobials. It has been suggested that increasing the ability of farmers to implement alternatives to antimicrobials will potentially enhance their efficacy on reducing AMU (42). Internal biosecurity has already been suggested as a potentially effective and feasible measure to lower AMU in pigs by European experts (43). Furthermore, field studies have concluded that improved internal biosecurity and management practices may be associated with reduced on-farm AMU (44–46). Using the all-in/all-out principle, conducting proper cleaning and disinfecting procedures, having a sickbay for diseased animals, and keeping animals of same age together are examples of good internal biosecurity practices that can reduce the spread of disease within the herd. An option to increase the level of biosecurity at national level within a sector is to require periodical systematic evaluations of biosecurity. This is currently in place in Denmark.

Control of illegal antimicrobial trade received low scores, mostly due to its limited feasibility. It is interesting to note that the ranked score for benchmarking strategies regarding AMU was higher in Denmark than in the other two countries. We hypothesize that this might be related to the successful implementation of the VetStat prescription database and the “Yellow card” benchmarking system (47, 48). In pigs, it should be highlighted that the “use of zinc” had the highest and the second highest score in Denmark and Portugal, respectively. This compound is often included in the feed, near the maximum allowed concentration, due to its positive effects on gastrointestinal health of pigs (49). The fact that no zinc oxide product is licensed in Switzerland might reflect why this measure only ranked 18th. Furthermore, it is likely that the total amounts of zinc oxide and the concentrations used in the feed throughout the production cycles vary between European countries. This may influence the effectiveness of this substance. In June 2017, the EU Commission decided to phase out the use of zinc oxide in all veterinary medical products over the next 5 years (50). This will put pressure on the pig production in Portugal and Denmark, and the need for prevention and alternative treatments will increase. Profiting from the sale of antimicrobials has been stressed as a possible conflict of interest (51). In Denmark, where veterinarians cannot make more than 5–10% profit from selling antimicrobials (52), the mean score of “limiting veterinarian’s profit from selling antimicrobials” was scored higher than in the other two countries. The perception of the Danish veterinarians might reflect the widespread discussion in the Danish veterinary community about AMU after banning the opportunity to profit from selling antimicrobials in 1995.

For Portuguese experts, “improve farm vaccination strategies” obtained the highest combined score for all livestock sectors except pigs (ranked 4th). This measure ranked much lower in Denmark and Switzerland, especially for broilers and dairy cattle. In an expert opinion conducted in Switzerland vaccines were perceived as effective but not important, which reflects the tendency not to vaccinate against livestock diseases in Switzerland (53). In Denmark, the KIK (Danish Quality Assurance) (54) has focused on improving biosecurity in broilers, which might have reduced the need for vaccines. For all livestock sectors investigated, the ranked scores for “improve farm vaccination strategies” were lower for Danish experts when compared with Portuguese or Swiss respondents. For pigs, the use of vaccination in Danish sow herds has been associated with a higher antimicrobial consumption in weaners compared with no use of vaccines against a specific disease agent. This might indicate that the occurrence of clinical disease is a driver for using vaccines in the first place (55, 56). Such usage patterns of vaccines might therefore explain the perception of Danish veterinarians that AMU in pigs could not be reduced through vaccination. Furthermore, due to the Danish specific pathogen-free system producers can verify the infection status of sow herds from which they intend to buy animals. This allows producers to buy animals from farms with a similar health status to their own, reducing the need for certain vaccinations (57). Postma et al. also detected an increased treatment incidence in pig herds that vaccinated for more pathogens. It was hypothesized that this finding was associated with a higher disease pressure in farms where infections were not yet under control through vaccination, flawed disease detection and/or factors related to the relationship between farmers and veterinarians (58).

It is also interesting to note that economic penalties related to antimicrobial consumption were not very well accepted by the participating veterinarians. Some respondents suggested offering incentives instead. Trade limitations were often scored as an effective measure, but difficult to implement. Scores related to on-farm treatment protocols and general veterinary treatment guidelines were contradictory, depending on the livestock sector and country, being generally scored lower in Portugal. In an European multi-country study, experts from the pig industry identified the development of clear on-farm action plans as a viable alternative to the use of antimicrobials (43). In another study, the implementation of treatment guidelines in Germany was followed by an increase in prudent AMU practices (59). In Denmark, treatment guidelines are in place for more than a decade, and in general the experience is that such guidelines help veterinarians to use the antimicrobials with highest effect on the infection and the lowest negative effect on resistance development (39).

The results obtained regarding the best interventions to reduce AMU are aligned with the existing literature. Postma et al. assessed the perceived effectiveness, feasibility and return of investment of a set of alternatives to AMU with veterinarians from six European countries. Overall, interventions that were ranked higher in the current study (e.g., internal biosecurity, increased vaccination, use of zinc, action plans) were also considered as the best alternatives in the study by Postma et al. (43).

The estimated potential for reduction varied largely between countries and livestock sectors. Danish estimates had a smaller variation for all livestock sectors, except for fattening calves. This might reflect the access of practitioners to the VetStat database. The detailed quantification of antimicrobial consumption per farm might allow veterinarians to have a better picture of what can indeed be achieved on the reduction of AMU. Furthermore, the potential of reduction needs to be interpreted taking into account the current level of antimicrobial consumption in each country/livestock sector (9). Visschers et al. conducted a questionnaire in six European countries and reported that Danish pig veterinarians were the ones that estimated the smallest potential for AMU reduction (42). This was also found in our study and might be related to the fact that antimicrobial sales in Denmark are low compared with other European countries (9). However, care should be taken when interpreting sales of antimicrobials in different countries as the potency of the antimicrobials used are not taken into consideration. Treatment incidence metrics can provide a more reliable assessment.

For certain combination of country/livestock sectors (e.g., broilers in Portugal or pigs in Switzerland), a high variability of answers was detected between the experts, which might be related to the personal experiences of the participating experts. It should be highlighted that the perceptions among the experts on AMU could have been influenced by on-going or recent country-specific media attention, surveillance programs, and interventions that have been discussed among veterinary professionals.

### Study Limitations

Our study has some potential biases that need to be acknowledged. Expert opinion is an accepted method when data are not available or cannot be easily obtained. Through our selection procedure, we tried to include veterinarians, who could provide a perspective of each country’s situation with regard to AMU. For the broiler sector it was not possible to achieve the recommended minimum number of five experts (16). However, it should be noted that due to the pyramidal and monopolized production of broilers in Europe, only few veterinarians are responsible for the largest part of a country’s production. Therefore, the veterinarians included in this study are highly specialized in poultry farming. Moreover, larger companies have standardized procedures with respect to antimicrobial treatments, so practitioners from the same companies would provide similar replies concerning AMU practices.

The fact that some experts were enrolled based on peer-recommendation might have introduced some bias into the selection procedure. Moreover, as it is often the case with expert opinions, the low sample size hinders the use of statistical methodologies to assess differences between countries.

It should be stressed that, the experts’ opinions on factors related to AST should not be completed disentangled; for instance, the importance of the time lag between sampling and getting the results can also be linked to the pressure exerted by the farmers and to an economic concern, as the risk of disease propagating within a herd increases with time. This should be taken into account when interpreting the results.

The proportion of treatment failures from 2005 to 2015 was investigated to obtain a picture on the direction of the trends during that time period. However, recall bias cannot be excluded in the estimates obtained for specific years, especially for 2005 and 2010. Furthermore, it must be stressed that a certain level of subjectivity exists, as the treatment failures might (as discussed earlier) be related to several factors such as wrong diagnosis or inappropriate treatment selection. This can bias the experts’ perception and influence the results obtained.

More attention should be given to the comparisons between the same livestock sectors in the different countries than to the overall results summarized per country or species. In these comparisons, some bias might have been introduced as the relative proportion of experts per country from each livestock sector varied slightly. In addition, the results for veal and fattening calves should be compared with care, given that differences exist between these two production types.

### Conclusion

Despite some observed variations between the individuals, countries and livestock sectors, the experts in this study acknowledged that there is potential for reducing AMU, particularly in the veal/fattening calf sector.

They agreed that mandatory official interventions are very likely to have a positive impact on reducing AMU in livestock.

In addition improved on-farm biosecurity measures (including internal biosecurity), education of veterinarians on prevention measures, early disease detection and rational AMU, vaccination and treatment plans were ranked high. The use of zinc oxide in pigs was also considered important, but will be phased out in EU.

The identified factors were mainly preventive measures. This highlights the importance of applying interventions that reduce the need for antimicrobials in the livestock industry. This paper was based on the key understanding of the issue by veterinarians that can inform and guide policymakers in Europe, the participating countries and other countries with a similar livestock production regarding their AMU policy.

## Ethics Statement

The approval by an ethics committee is not required for this type of study. The objectives of the study were communicated to all participants by electronic mail before they reply the questionnaire. Participants were also informed that their data would be used for a scientific publication. All experts were assured anonymity.

## Author Contributions

All the authors were involved in the conceptualization of the paper. LC drafted the questionnaire, which was review and approved by all the other authors. Data management and data analysis were performed by LC. IM, GR, and LN were involved in the funding acquisition. IM and LN were responsible for the doctoral candidate (LC) supervision. LC drafted the paper, which was reviewed and approved by all the authors.

## Conflict of Interest Statement

The authors declare that the research was conducted in the absence of any commercial or financial relationships that could be construed as a potential conflict of interest.

## Supplementary Material

Figure S1 Factors influencing the use of antimicrobial sensitivity testing before antimicrobial prescription. Veterinary experts (*n* = 67) were asked score from 1 (low importance) to 10 (high importance) potential reasons for not conducting antimicrobial susceptibility testing (AST) more often. Colour dots represent individual answers from experts of different livestock sectors. The violin plot represents the probability density at each score value. ST_time_lag—“Time lag between sampling and obtaining the result”; ST_not_reliable—“Lack of reliability of the method”; ST_cost—“Cost/Price/Economic reasons”; ST_not_help—“Antimicrobial susceptibility testing results do not help on the clinical decision making process (or on the selection of the antimicrobial to be used)”; ST_farmer_pressure—“Farmer’s pressure to have an immediate treatment for the animals”; ST_lack_lab—“Lack of laboratories providing the service/Laboratories unable to provide an answer to the demand.” *n*, number of answers.

Figure S2 Antimicrobial classes that most frequently lead to treatment failures. Veterinary experts (*n* = 67) were asked to mention up to three antimicrobial classes that most frequently lead to treatment failures. Results were stratified per country and livestock sector. *n*, number of answers.

Figure S3 Antimicrobial classes for which the proportion of treatment failures increased the most between 2005 and 2015. Veterinary experts (*n* = 67) were asked to mention up to three antimicrobial classes for which the proportion of treatment failures increased the most between 2005 and 2015. Results were stratified per country and livestock sector. *n*, number of answers.
