# Supplementary figures and images for: Veterinary Expert Opinion on Potential Drivers and Opportunities for Changing Antimicrobial Usage Practices in Livestock in Denmark, Portugal, and Switzerland
Source: Front Vet Sci. 2018 Mar 1;5:29. doi: 10.3389/fvets.2018.00029 (PMC5837977; doi:10.3389/fvets.2018.00029)

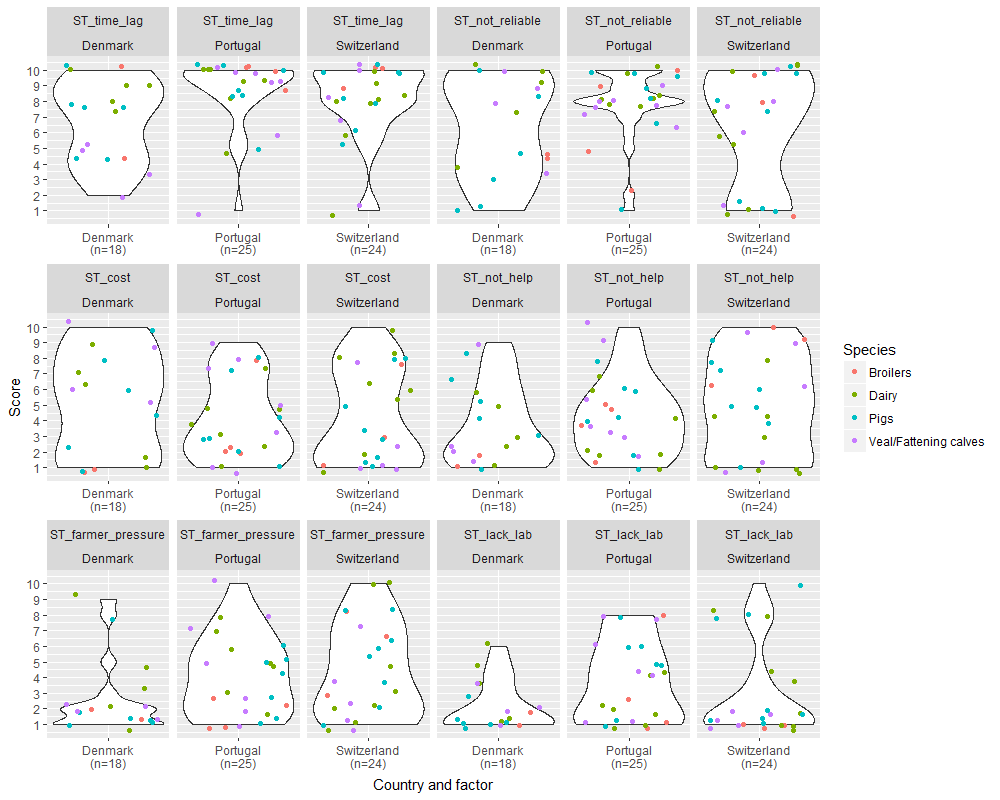

Supplement: Figure S1 — Factors influencing the use of antimicrobial sensitivity testing before antimicrobial prescription. Veterinary experts (n = 67) were asked score from 1 (low importance) to 10 (high importance) potential reasons for not conducting antimicrobial susceptibility testing (AST) more often. Colour dots represent individual answers from experts of different livestock sectors. The violin plot represents the probability density at each score value. ST_time_lag—“Time lag between sampling and obtaining the result”; ST_not_reliable—“Lack of reliability of the method”; ST_cost—“Cost/Price/Economic reasons”; ST_not_help—“Antimicrobial susceptibility testing results do not help on the clinical decision making process (or on the selection of the antimicrobial to be used)”; ST_farmer_pressure—“Farmer’s pressure to have an immediate treatment for the animals”; ST_lack_lab—“Lack of laboratories providing the service/Laboratories unable to provide an answer to the demand.” n, number of answers. [file image_1.tiff]

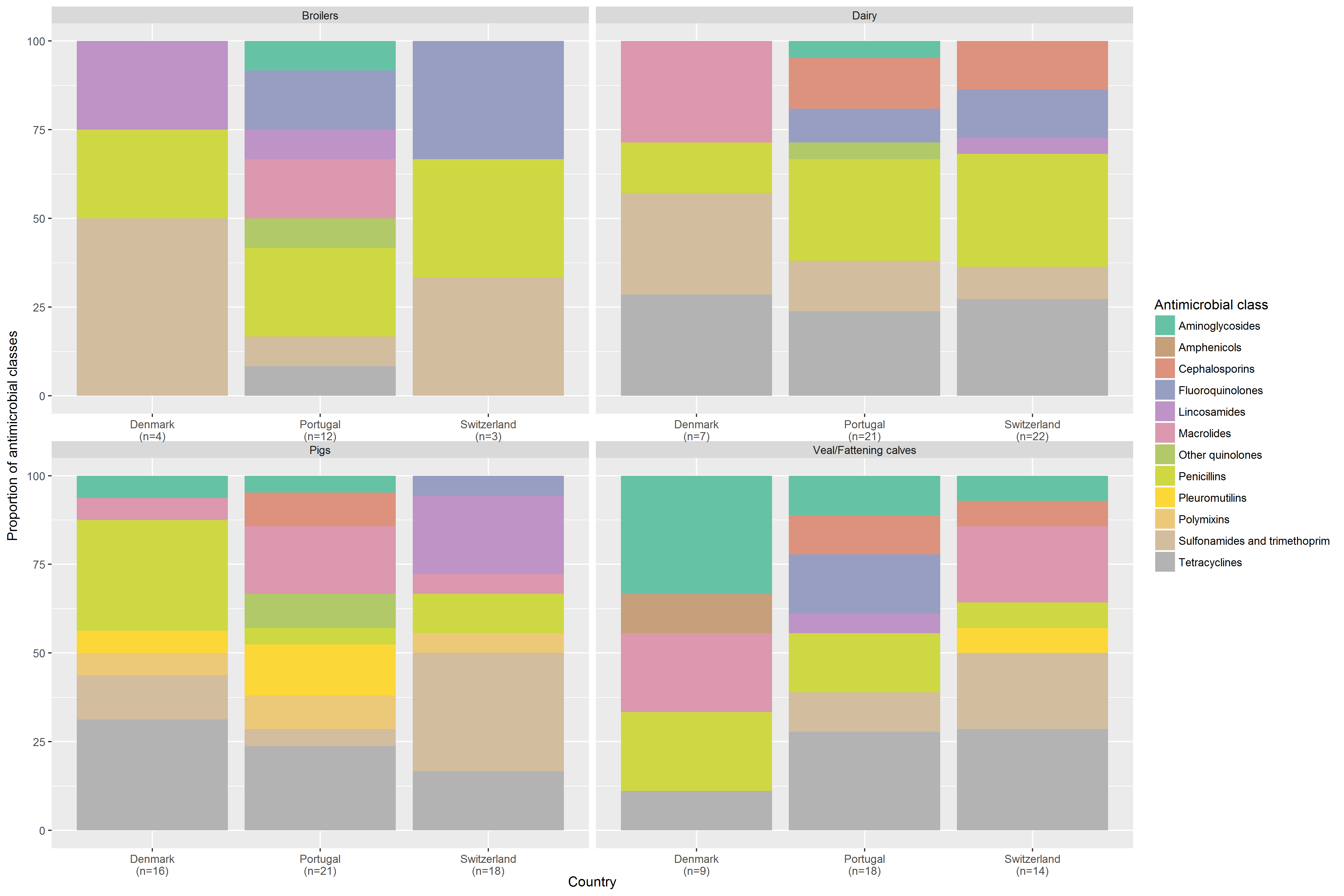

Supplement: Figure S2 — Antimicrobial classes that most frequently lead to treatment failures. Veterinary experts (n = 67) were asked to mention up to three antimicrobial classes that most frequently lead to treatment failures. Results were stratified per country and livestock sector. n, number of answers. [file image_2.tiff]

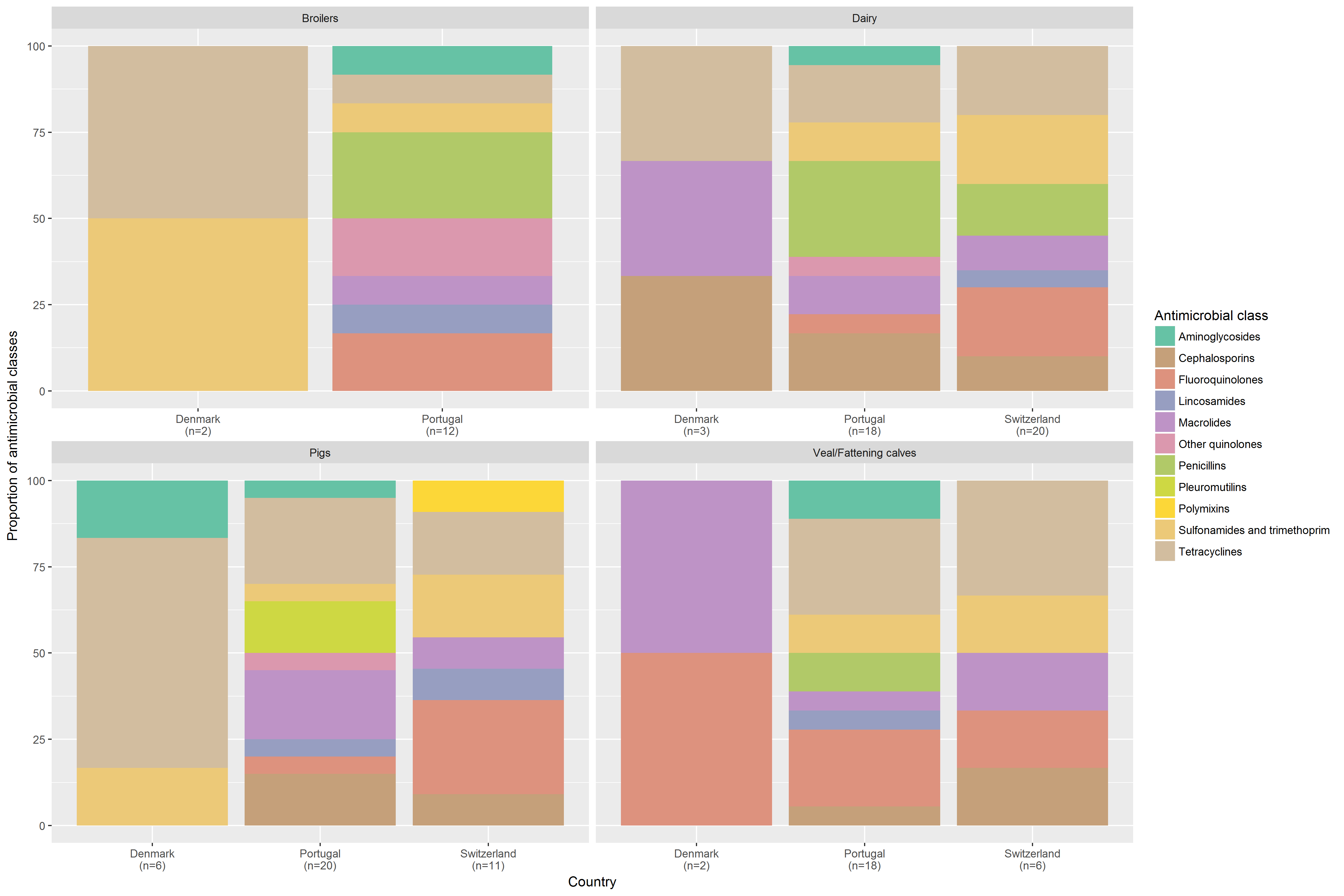

Supplement: Figure S3 — Antimicrobial classes for which the proportion of treatment failures increased the most between 2005 and 2015. Veterinary experts (n = 67) were asked to mention up to three antimicrobial classes for which the proportion of treatment failures increased the most between 2005 and 2015. Results were stratified per country and livestock sector. n, number of answers. [file image_3.tiff]
