# Supplementary material for: Veterinary Expert Opinion on Potential Drivers and Opportunities for Changing Antimicrobial Usage Practices in Livestock in Denmark, Portugal, and Switzerland
Source: Front Vet Sci. 2018 Mar 1;5:29. doi: 10.3389/fvets.2018.00029 (PMC5837977; doi:10.3389/fvets.2018.00029)
Supplement: Supplementary file 5 [file data_sheet_2.docx]

| Supplementary material 2. Number of participating veterinary experts stratified per country and livestock sector. | | | | | | | | | |  |  |  |  |
| --- | --- | --- | --- | --- | --- | --- | --- | --- | --- | --- | --- | --- | --- |
|  | Denmark | | | Portugal | | | Switzerland | | | | Total | | |
|  | n | Affiliated to academia (%) | Median years of experience | n | Affiliated to academia (%) | Median years of experience | n | Affiliated to academia (%) | Median years of experience | | n | Affiliated to academia (%) | Median years of experience |
| Broilers | 3 | 0% | 3 | 4 | 0% | 17 | 3 | 0% | 9 | | 10 | 0% | 10.5 |
| Pigs | 6 | 0% | 15 | 7 | 0% | 16 | 8 | 37.5% | 20 | | 21 | 14% | 20 |
| Dairy cattle | 5 | 0% | 27 | 7 | 43% | 16 | 8 | 37.5% | 12.5 | | 20 | 30% | 15.5 |
| Veal/Fattening calves | 4 | 0% | 17.5 | 7 | 43% | 20 | 5 | 20% | 25 | | 16 | 25% | 20 |
| Total | 18 | 0% | 15 | 25 | 24% | 17 | 24 | 29% | 20 | | 67 | 19% | 17 |
