# Supplementary material for: Veterinary Expert Opinion on Potential Drivers and Opportunities for Changing Antimicrobial Usage Practices in Livestock in Denmark, Portugal, and Switzerland
Source: Front Vet Sci. 2018 Mar 1;5:29. doi: 10.3389/fvets.2018.00029 (PMC5837977; doi:10.3389/fvets.2018.00029)
Supplement: Supplementary file 6 [file data_sheet_3.docx]

| Impact and feasibility of interventions to reduce antimicrobial use per country | | | | | | | | | | | | | | | | | | |
| --- | --- | --- | --- | --- | --- | --- | --- | --- | --- | --- | --- | --- | --- | --- | --- | --- | --- | --- |
|  | Switzerland | | | | | | Denmark | | | | | | Portugal | | | | | |
|  | Score | | | Feasibility | Impact | n | Score | | | Feasibility | Impact | n | Score | | | Feasibility | Impact | n |
| variable | Rank | Mean | SD | Mean | Mean |  | Rank | Mean | SD | Mean | Mean |  | Rank | Mean | SD | Mean | Mean |  |
| vac | 1 | 7.7 | 1.9 | 7.0 | 7.4 | 23 | 14 | 5.5 | 1.7 | 5.6 | 5.5 | 18 | 2 | 8.2 | 2.0 | 7.8 | 8.0 | 25 |
| eduv | 2 | 7.4 | 1.9 | 7.8 | 7.6 | 23 | 6 | 6.7 | 2.0 | 6.4 | 6.5 | 18 | 3 | 7.4 | 2.0 | 7.6 | 7.5 | 25 |
| txplan | 2 | 7.4 | 1.8 | 7.4 | 7.4 | 23 | 3 | 7.4 | 2.6 | 5.2 | 6.3 | 18 | 4 | 7.2 | 2.3 | 7.5 | 7.4 | 25 |
| intbio | 4 | 7.2 | 2.3 | 8.4 | 7.8 | 23 | 9 | 6.2 | 2.2 | 8.3 | 7.2 | 18 | 6 | 6.4 | 2.0 | 8.9 | 7.6 | 25 |
| guide | 5 | 7.0 | 2.1 | 5.9 | 6.5 | 23 | 4 | 7.3 | 2.4 | 5.3 | 6.3 | 18 | 10 | 5.9 | 3.1 | 5.8 | 5.9 | 25 |
| probio | 6 | 6.7 | 2.6 | 4.5 | 5.6 | 23 | 20 | 3.9 | 2.7 | 3.4 | 3.7 | 18 | 8 | 6.0 | 2.9 | 5.6 | 5.8 | 25 |
| water | 6 | 6.7 | 2.5 | 5.9 | 6.3 | 23 | 13 | 5.6 | 2.3 | 4.8 | 5.2 | 18 | 5 | 6.6 | 2.2 | 7.1 | 6.8 | 25 |
| eduf | 8 | 6.1 | 2.1 | 8.4 | 7.3 | 23 | 12 | 5.9 | 2.4 | 7.0 | 6.4 | 18 | 11 | 5.8 | 2.3 | 8.0 | 6.9 | 25 |
| extbio | 10 | 5.8 | 2.7 | 7.0 | 6.4 | 23 | 11 | 6.0 | 1.9 | 6.6 | 6.3 | 18 | 17 | 5.1 | 1.9 | 7.8 | 6.5 | 25 |
| profit | 10 | 5.8 | 2.5 | 4.2 | 5.0 | 23 | 2 | 7.5 | 3.9 | 6.0 | 6.8 | 18 | 20 | 4.8 | 3.3 | 4.3 | 4.6 | 24 |
| dxpath | 11 | 5.6 | 2.3 | 7.3 | 6.5 | 23 | 19 | 4.4 | 1.9 | 6.6 | 5.5 | 18 | 15 | 5.4 | 2.3 | 7.3 | 6.3 | 25 |
| ban | 12 | 5.5 | 3.0 | 5.2 | 5.4 | 23 | 8 | 6.6 | 3.0 | 5.2 | 5.9 | 18 | 10 | 5.9 | 3.0 | 6.2 | 6.0 | 24 |
| feed | 12 | 5.5 | 2.3 | 6.4 | 6.0 | 23 | 16 | 5.4 | 2.3 | 7.1 | 6.2 | 18 | 8 | 6.0 | 1.9 | 7.2 | 6.6 | 25 |
| label | 14 | 5.3 | 2.4 | 5.3 | 5.3 | 23 | 6 | 6.7 | 2.4 | 4.4 | 5.6 | 18 | 12 | 5.7 | 2.7 | 5.6 | 5.6 | 25 |
| st | 15 | 5.2 | 2.5 | 6.2 | 5.7 | 23 | 17 | 4.8 | 2.2 | 5.3 | 5.1 | 18 | 19 | 4.9 | 1.9 | 6.8 | 5.8 | 25 |
| econ | 16 | 5.0 | 2.2 | 7.0 | 6.0 | 23 | 16 | 5.4 | 3.0 | 6.9 | 6.2 | 18 | 16 | 5.2 | 2.9 | 7.1 | 6.1 | 25 |
| trade | 16 | 5.0 | 2.3 | 6.9 | 5.9 | 23 | 18 | 4.7 | 2.7 | 8.1 | 6.4 | 18 | 18 | 5.0 | 2.9 | 7.2 | 6.1 | 25 |
| zinc | 18 | 4.9 | 4.3 | 5.5 | 5.2 | 23 | 1 | 9.0 | 1.3 | 8.5 | 8.8 | 6 | 1 | 8.4 | 2.0 | 7.7 | 8.1 | 7 |
| benchf | 20 | 4.6 | 2.3 | 6.7 | 5.6 | 23 | 6 | 6.7 | 2.3 | 7.1 | 6.9 | 18 | 12 | 5.7 | 2.9 | 6.8 | 6.3 | 25 |
| dens | 20 | 4.6 | 2.3 | 7.8 | 6.2 | 23 | 22 | 3.5 | 2.2 | 6.9 | 5.2 | 18 | 14 | 5.6 | 2.2 | 7.4 | 6.5 | 25 |
| benchv | 21 | 4.0 | 2.1 | 5.8 | 4.9 | 23 | 10 | 6.1 | 2.6 | 6.5 | 6.3 | 18 | 20 | 4.8 | 2.6 | 6.4 | 5.6 | 25 |

| Impact and feasibility of interventions to reduce antimicrobial use in the broiler sector | | | | | | | | | | | | | | | | | | |
| --- | --- | --- | --- | --- | --- | --- | --- | --- | --- | --- | --- | --- | --- | --- | --- | --- | --- | --- |
|  | Switzerland | | | | | | Denmark | | | | | | Portugal | | | | | |
|  | Score | | | Feasibility | Impact | n | Score | | | Feasibility | Impact | n | Score | | | Feasibility | Impact | n |
| variable | Rank | Mean | SD | Mean | Mean |  | Rank | Mean | SD | Mean | Mean |  | Rank | Mean | SD | Mean | Mean |  |
| eduv | 2 | 8.0 | 0.7 | 8.0 | 8.0 | 2 | 7 | 6.0 | 2.8 | 5.7 | 6.3 | 3 | 3 | 8.5 | 1.0 | 8.5 | 8.5 | 4 |
| intbio | 2 | 8.0 | 0.7 | 7.5 | 8.5 | 2 | 14 | 5.0 | 1.3 | 3.7 | 6.3 | 3 | 4 | 8.4 | 1.4 | 7.5 | 9.2 | 4 |
| dens | 4 | 7.8 | 0.4 | 7.0 | 8.5 | 2 | 19 | 3.5 | 1.8 | 1.3 | 5.7 | 3 | 15 | 5.9 | 1.6 | 4.0 | 7.8 | 4 |
| eduf | 4 | 7.8 | 0.4 | 7.0 | 8.5 | 2 | 12 | 5.3 | 2.0 | 5.0 | 5.7 | 3 | 6 | 7.4 | 1.5 | 6.0 | 8.8 | 4 |
| feed | 6 | 7.2 | 0.4 | 6.5 | 8.0 | 2 | 12 | 5.3 | 1.3 | 2.7 | 8.0 | 3 | 9 | 6.9 | 1.0 | 6.0 | 7.8 | 4 |
| guide | 6 | 7.2 | 0.4 | 8.0 | 6.5 | 2 | 4 | 6.5 | 4.8 | 6.7 | 6.3 | 3 | 21 | 3.2 | 3.2 | 3.2 | 3.2 | 4 |
| probio | 6 | 7.2 | 1.1 | 8.0 | 6.5 | 2 | 20 | 2.7 | 0.6 | 1.7 | 3.7 | 3 | 5 | 8.0 | 1.4 | 8.8 | 7.2 | 4 |
| water | 6 | 7.2 | 0.4 | 6.5 | 8.0 | 2 | 6 | 6.2 | 1.5 | 4.7 | 7.7 | 3 | 2 | 8.6 | 1.1 | 8.8 | 8.5 | 4 |
| txplan | 10 | 6.8 | 1.8 | 6.5 | 7.0 | 2 | 16 | 4.7 | 1.4 | 7.7 | 1.7 | 3 | 8 | 7.1 | 2.3 | 7.2 | 7.0 | 4 |
| vac | 10 | 6.8 | 1.8 | 6.5 | 7.0 | 2 | 13 | 5.2 | 2.0 | 4.3 | 6.0 | 3 | 1 | 9.0 | 1.2 | 8.5 | 9.5 | 4 |
| dxpath | 12 | 6.5 | 2.1 | 6.0 | 7.0 | 2 | 16 | 4.7 | 2.5 | 2.3 | 7.0 | 3 | 10 | 6.8 | 2.1 | 5.8 | 7.8 | 4 |
| econ | 12 | 6.5 | 2.1 | 6.5 | 6.5 | 2 | 1 | 8.2 | 1.8 | 7.7 | 8.7 | 3 | 12 | 6.4 | 2.7 | 5.2 | 7.5 | 4 |
| illegal | 13 | 6.2 | 1.1 | 7.5 | 5.0 | 2 | 18 | 4.0 | 1.3 | 1.7 | 6.3 | 3 | 12 | 6.4 | 1.5 | 5.0 | 7.8 | 4 |
| extbio | 14 | 6.0 | NA | 6.0 | 6.0 | 1 | 10 | 5.5 | 0.9 | 4.0 | 7.0 | 3 | 8 | 7.1 | 0.9 | 5.2 | 9.0 | 4 |
| label | 16 | 5.2 | 2.5 | 5.0 | 5.5 | 2 | 9 | 5.7 | 1.3 | 6.7 | 4.7 | 3 | 18 | 5.4 | 3.4 | 5.8 | 5.0 | 4 |
| profit | 16 | 5.2 | 4.6 | 6.0 | 4.5 | 2 | 21 | 2.5 | 2.6 | 4.0 | 1.0 | 3 | 20 | 4.2 | 3.9 | 4.2 | 4.2 | 4 |
| trade | 16 | 5.2 | 1.8 | 5.0 | 5.5 | 2 | 2 | 7.8 | 1.9 | 7.0 | 8.7 | 3 | 16 | 5.6 | 1.9 | 4.5 | 6.8 | 4 |
| benchf | 18 | 4.8 | 1.1 | 4.0 | 5.5 | 2 | 5 | 6.3 | 1.8 | 6.7 | 6.0 | 3 | 12 | 6.4 | 3.3 | 6.2 | 6.5 | 4 |
| st | 18 | 4.8 | 1.1 | 4.0 | 5.5 | 2 | 18 | 4.0 | 2.6 | 2.3 | 5.7 | 3 | 14 | 6.2 | 1.8 | 5.0 | 7.5 | 4 |
| benchv | 20 | 4.2 | 2.5 | 3.5 | 5.0 | 2 | 8 | 5.8 | 2.6 | 6.3 | 5.3 | 3 | 16 | 5.6 | 2.3 | 4.5 | 6.8 | 4 |
| ban | 21 | 3.8 | 1.8 | 3.5 | 4.0 | 2 | 2 | 7.8 | 2.3 | 9.3 | 6.3 | 3 | 19 | 4.8 | 2.4 | 4.8 | 4.8 | 4 |

| Impact and feasibility of interventions to reduce antimicrobial use in the dairy cattle sector | | | | | | | | | | | | | | | | | | |
| --- | --- | --- | --- | --- | --- | --- | --- | --- | --- | --- | --- | --- | --- | --- | --- | --- | --- | --- |
|  | Switzerland | | | | | | Denmark | | | | | | Portugal | | | | | |
|  | Score | | | Feasibility | Impact | n | Score | | | Feasibility | Impact | n | Score | | | Feasibility | Impact | n |
| variable | Rank | Mean | SD | Mean | Mean |  | Rank | Mean | SD | Mean | Mean |  | Rank | Mean | SD | Mean | Mean |  |
| intbio | 1 | 8.0 | 1.9 | 6.9 | 9.1 | 8 | 4 | 7.4 | 1.2 | 6.2 | 8.6 | 5 | 2 | 7.4 | 1.1 | 5.6 | 9.1 | 7 |
| eduv | 2 | 7.7 | 1.6 | 7.4 | 8.0 | 8 | 4 | 7.4 | 1.2 | 6.8 | 8.0 | 5 | 2 | 7.4 | 1.2 | 5.9 | 8.9 | 7 |
| txplan | 3 | 7.6 | 1.8 | 7.6 | 7.6 | 8 | 12 | 5.9 | 2.7 | 5.8 | 6.0 | 5 | 4 | 7.3 | 1.7 | 5.9 | 8.7 | 7 |
| eduf | 4 | 7.3 | 1.9 | 6.0 | 8.6 | 8 | 8 | 6.7 | 2.3 | 6.2 | 7.2 | 5 | 6 | 6.6 | 1.7 | 5.0 | 8.3 | 7 |
| dxpath | 6 | 7.2 | 1.6 | 6.9 | 7.5 | 8 | 14 | 5.6 | 0.7 | 3.8 | 7.4 | 5 | 13 | 5.8 | 1.3 | 4.6 | 7.0 | 7 |
| water | 6 | 7.2 | 1.8 | 8.0 | 6.4 | 8 | 20 | 4.3 | 2.2 | 5.2 | 3.4 | 5 | 11 | 5.9 | 2.7 | 5.6 | 6.1 | 7 |
| guide | 7 | 6.9 | 1.7 | 6.9 | 6.9 | 8 | 5 | 7.0 | 1.2 | 6.8 | 7.2 | 5 | 5 | 7.0 | 2.1 | 6.1 | 7.9 | 7 |
| st | 8 | 6.4 | 2.1 | 6.5 | 6.4 | 8 | 15 | 5.1 | 0.7 | 5.4 | 4.8 | 5 | 11 | 5.9 | 1.4 | 4.7 | 7.0 | 7 |
| feed | 9 | 6.4 | 2.0 | 5.9 | 6.9 | 8 | 8 | 6.7 | 1.7 | 6.2 | 7.2 | 5 | 19 | 5.1 | 1.7 | 4.1 | 6.0 | 7 |
| vac | 10 | 6.3 | 1.9 | 6.4 | 6.2 | 8 | 17 | 4.7 | 1.3 | 4.8 | 4.6 | 5 | 1 | 7.6 | 2.1 | 7.1 | 8.1 | 7 |
| dens | 11 | 6.2 | 2.3 | 5.1 | 7.4 | 8 | 13 | 5.8 | 2.3 | 5.2 | 6.4 | 5 | 14 | 5.7 | 1.8 | 4.6 | 6.9 | 7 |
| ban | 12 | 5.9 | 1.3 | 6.4 | 5.5 | 8 | 10 | 6.5 | 3.7 | 5.6 | 7.4 | 5 | 8 | 6.0 | 2.4 | 5.2 | 7.0 | 6 |
| extbio | 13 | 5.8 | 2.7 | 4.7 | 6.9 | 7 | 16 | 5.0 | 2.0 | 5.2 | 4.8 | 5 | 16 | 5.3 | 1.7 | 4.7 | 5.9 | 7 |
| econ | 14 | 5.8 | 1.6 | 4.9 | 6.6 | 8 | 11 | 6.0 | 1.7 | 5.0 | 7.0 | 5 | 11 | 5.9 | 2.3 | 4.3 | 7.4 | 7 |
| trade | 15 | 5.6 | 1.8 | 4.2 | 6.9 | 8 | 10 | 6.5 | 2.0 | 4.0 | 9.0 | 5 | 7 | 6.1 | 2.1 | 4.3 | 7.9 | 7 |
| label | 16 | 5.4 | 1.5 | 5.8 | 5.1 | 8 | 18 | 4.5 | 1.7 | 4.4 | 4.6 | 5 | 17 | 5.2 | 2.2 | 4.0 | 6.4 | 7 |
| benchv | 17 | 5.0 | 2.1 | 4.1 | 5.9 | 8 | 6 | 6.8 | 1.2 | 6.0 | 7.6 | 5 | 18 | 5.1 | 2.8 | 4.1 | 6.1 | 7 |
| benchf | 18 | 4.9 | 2.1 | 4.1 | 5.8 | 8 | 2 | 7.5 | 1.6 | 7.0 | 8.0 | 5 | 8 | 6.0 | 2.3 | 4.9 | 7.1 | 7 |
| profit | 18 | 4.9 | 1.5 | 5.2 | 4.6 | 8 | 1 | 8.3 | 3.0 | 8.2 | 8.4 | 5 | 20 | 4.2 | 3.4 | 4.7 | 3.7 | 6 |
| illegal | 20 | 4.6 | 2.6 | 3.6 | 5.6 | 8 | 18 | 4.5 | 1.2 | 2.8 | 6.2 | 5 | 15 | 5.6 | 2.0 | 2.9 | 8.3 | 7 |
| probio | 21 | 4.5 | 2.4 | 5.8 | 3.2 | 8 | 21 | 2.8 | 1.6 | 2.8 | 2.8 | 5 | 21 | 3.4 | 1.7 | 2.4 | 4.4 | 7 |

| Impact and feasibility of interventions to reduce antimicrobial use in the pig sector | | | | | | | | | | | | | | | | | | |
| --- | --- | --- | --- | --- | --- | --- | --- | --- | --- | --- | --- | --- | --- | --- | --- | --- | --- | --- |
|  | Switzerland | | | | | | Denmark | | | | | | Portugal | | | | | |
|  | Score | | | Feasibility | Impact | n | Score | | | Feasibility | Impact | n | Score | | | Feasibility | Impact | n |
| variable | Rank | Mean | SD | Mean | Mean |  | Rank | Mean | SD | Mean | Mean |  | Rank | Mean | SD | Mean | Mean |  |
| vac | 1 | 8.8 | 1.3 | 9.1 | 8.4 | 8 | 7 | 7.0 | 1.6 | 6.8 | 7.2 | 6 | 4 | 7.9 | 1.2 | 8.7 | 7.1 | 7 |
| intbio | 2 | 8.4 | 1.0 | 8.1 | 8.6 | 8 | 2 | 8.2 | 1.3 | 7.8 | 8.7 | 6 | 1 | 8.3 | 0.8 | 7.4 | 9.1 | 7 |
| txplan | 3 | 7.7 | 1.9 | 7.8 | 7.6 | 8 | 5 | 7.2 | 2.1 | 8.5 | 5.8 | 6 | 6 | 7.6 | 1.1 | 8.6 | 6.7 | 7 |
| extbio | 4 | 7.2 | 0.9 | 6.9 | 7.5 | 8 | 4 | 7.2 | 1.3 | 7.3 | 7.2 | 6 | 5 | 7.8 | 1.0 | 6.3 | 9.3 | 7 |
| eduv | 5 | 7.1 | 1.4 | 6.8 | 7.5 | 8 | 10 | 6.3 | 1.3 | 6.8 | 5.8 | 6 | 7 | 7.4 | 1.3 | 8.0 | 6.9 | 7 |
| trade | 6 | 7.1 | 1.8 | 5.4 | 8.8 | 8 | 14 | 6.2 | 2.2 | 5.0 | 7.3 | 6 | 14 | 6.4 | 2.8 | 6.1 | 6.7 | 7 |
| eduf | 7 | 7.0 | 1.1 | 5.2 | 8.8 | 8 | 8 | 6.9 | 1.2 | 7.0 | 6.8 | 6 | 12 | 6.9 | 1.5 | 6.4 | 7.4 | 7 |
| benchf | 8 | 6.9 | 2.0 | 5.1 | 8.8 | 8 | 6 | 7.1 | 1.6 | 6.8 | 7.3 | 6 | 16 | 6.3 | 2.8 | 5.7 | 6.9 | 7 |
| econ | 8 | 6.9 | 1.7 | 5.0 | 8.9 | 8 | 19 | 5.5 | 1.3 | 5.7 | 5.3 | 6 | 17 | 6.2 | 2.9 | 5.7 | 6.7 | 7 |
| water | 10 | 6.9 | 1.6 | 6.8 | 7.0 | 8 | 12 | 6.2 | 1.3 | 6.8 | 5.7 | 6 | 11 | 7.1 | 1.4 | 6.9 | 7.3 | 7 |
| dxpath | 12 | 6.6 | 2.0 | 4.9 | 8.4 | 8 | 15 | 5.8 | 1.3 | 5.3 | 6.3 | 6 | 10 | 7.3 | 1.3 | 6.7 | 7.9 | 7 |
| probio | 12 | 6.6 | 1.4 | 7.5 | 5.8 | 8 | 17 | 5.8 | 2.2 | 6.8 | 4.7 | 6 | 8 | 7.4 | 0.9 | 8.0 | 6.7 | 7 |
| feed | 13 | 6.5 | 1.4 | 5.9 | 7.1 | 8 | 9 | 6.5 | 1.1 | 5.7 | 7.3 | 6 | 8 | 7.4 | 0.9 | 6.9 | 7.9 | 7 |
| guide | 14 | 6.4 | 1.6 | 7.9 | 4.9 | 8 | 17 | 5.8 | 0.4 | 7.5 | 4.0 | 6 | 12 | 6.9 | 1.7 | 7.7 | 6.1 | 7 |
| st | 15 | 6.1 | 2.1 | 5.0 | 7.1 | 8 | 20 | 5.3 | 1.5 | 5.3 | 5.3 | 6 | 16 | 6.3 | 0.8 | 5.6 | 7.0 | 7 |
| dens | 16 | 5.9 | 1.8 | 3.8 | 8.1 | 8 | 14 | 6.2 | 1.2 | 4.2 | 8.2 | 6 | 4 | 7.9 | 1.2 | 7.4 | 8.4 | 7 |
| profit | 17 | 5.3 | 0.7 | 6.4 | 4.2 | 8 | 3 | 8.2 | 3.6 | 8.2 | 8.2 | 6 | 22 | 4.9 | 2.6 | 5.4 | 4.4 | 7 |
| benchv | 18 | 5.2 | 2.5 | 3.8 | 6.6 | 8 | 10 | 6.3 | 2.1 | 6.5 | 6.2 | 6 | 20 | 5.5 | 2.4 | 5.1 | 5.9 | 7 |
| zinc | 18 | 5.2 | 2.3 | 4.9 | 5.5 | 8 | 1 | 8.8 | 1.3 | 9.0 | 8.5 | 6 | 2 | 8.1 | 1.9 | 8.4 | 7.7 | 7 |
| ban | 20 | 5.1 | 2.1 | 5.2 | 5.0 | 8 | 22 | 4.1 | 1.8 | 5.8 | 2.3 | 6 | 18 | 5.9 | 1.2 | 6.7 | 5.1 | 7 |
| label | 21 | 4.9 | 2.1 | 4.2 | 5.6 | 8 | 17 | 5.8 | 1.6 | 7.2 | 4.3 | 6 | 19 | 5.7 | 2.8 | 6.3 | 5.1 | 7 |
| illegal | 22 | 4.1 | 2.1 | 3.4 | 4.9 | 8 | 22 | 4.1 | 2.3 | 4.7 | 3.5 | 6 | 21 | 5.4 | 2.5 | 4.7 | 6.1 | 7 |

| Impact and feasibility of interventions to reduce antimicrobial use in the veal/fattening calf sector | | | | | | | | | | | | | | | | | | |
| --- | --- | --- | --- | --- | --- | --- | --- | --- | --- | --- | --- | --- | --- | --- | --- | --- | --- | --- |
|  | Switzerland | | | | | | Denmark | | | | | | Portugal | | | | | |
|  | Score | | | Feasibility | Impact | n | Score | | | Feasibility | Impact | n | Score | | | Feasibility | Impact | n |
| variable | Rank | Mean | SD | Mean | Mean |  | Rank | Mean | SD | Mean | Mean |  | Rank | Mean | SD | Mean | Mean |  |
| eduv | 1 | 8.0 | 1.5 | 8.2 | 7.8 | 5 | 8 | 6.1 | 0.9 | 7.0 | 5.2 | 4 | 4 | 7.1 | 1.4 | 7.6 | 6.7 | 7 |
| eduf | 2 | 7.4 | 1.9 | 7.4 | 7.4 | 5 | 6 | 6.2 | 1.2 | 4.5 | 8.0 | 4 | 5 | 6.9 | 2.0 | 5.9 | 8.0 | 7 |
| vac | 3 | 7.1 | 2.0 | 8.2 | 6.0 | 5 | 17 | 4.6 | 1.2 | 5.2 | 4.0 | 4 | 1 | 7.9 | 1.2 | 8.4 | 7.3 | 7 |
| txplan | 4 | 6.9 | 2.2 | 6.8 | 7.0 | 5 | 4 | 6.6 | 0.9 | 7.5 | 5.8 | 4 | 2 | 7.3 | 1.0 | 7.3 | 7.3 | 7 |
| intbio | 5 | 6.5 | 1.8 | 6.2 | 6.8 | 5 | 1 | 7.2 | 1.3 | 5.8 | 8.8 | 4 | 6 | 6.8 | 0.9 | 5.4 | 8.1 | 7 |
| extbio | 6 | 6.1 | 2.2 | 5.6 | 6.6 | 5 | 2 | 7.1 | 1.1 | 6.5 | 7.8 | 4 | 16 | 5.9 | 1.2 | 4.3 | 7.6 | 7 |
| dens | 7 | 5.8 | 0.8 | 4.0 | 7.6 | 5 | 19 | 4.2 | 1.6 | 2.0 | 6.5 | 4 | 12 | 6.1 | 1.5 | 5.6 | 6.7 | 7 |
| guide | 8 | 5.7 | 2.3 | 5.6 | 5.8 | 5 | 8 | 6.1 | 0.5 | 8.0 | 4.2 | 4 | 20 | 5.1 | 1.2 | 5.4 | 4.9 | 7 |
| label | 9 | 5.6 | 2.5 | 6.2 | 5.0 | 5 | 4 | 6.6 | 1.8 | 9.0 | 4.2 | 4 | 12 | 6.1 | 0.9 | 6.9 | 5.4 | 7 |
| ban | 10 | 5.5 | 2.6 | 5.4 | 5.6 | 5 | 5 | 6.4 | 1.8 | 6.8 | 6.0 | 4 | 6 | 6.8 | 1.7 | 6.4 | 7.1 | 7 |
| benchf | 12 | 5.0 | 2.4 | 4.6 | 5.4 | 5 | 8 | 6.1 | 2.4 | 6.0 | 6.2 | 4 | 8 | 6.5 | 2.5 | 6.3 | 6.7 | 7 |
| dxpath | 12 | 5.0 | 1.9 | 4.6 | 5.4 | 5 | 16 | 5.4 | 0.9 | 5.2 | 5.5 | 4 | 17 | 5.6 | 2.4 | 4.6 | 6.7 | 7 |
| probio | 12 | 5.0 | 2.5 | 6.4 | 3.6 | 5 | 21 | 2.5 | 1.2 | 2.8 | 2.2 | 4 | 18 | 5.5 | 1.5 | 6.1 | 4.9 | 7 |
| trade | 12 | 5.0 | 2.5 | 5.4 | 4.6 | 5 | 14 | 5.5 | 2.4 | 3.5 | 7.5 | 4 | 14 | 6.1 | 2.2 | 5.0 | 7.1 | 7 |
| econ | 15 | 4.6 | 2.1 | 4.4 | 4.8 | 5 | 12 | 5.9 | 2.2 | 3.8 | 8.0 | 4 | 11 | 6.2 | 1.5 | 5.4 | 7.0 | 7 |
| benchv | 16 | 4.5 | 2.5 | 4.4 | 4.6 | 5 | 10 | 6.0 | 2.5 | 5.5 | 6.5 | 4 | 14 | 6.1 | 1.6 | 5.1 | 7.0 | 7 |
| profit | 16 | 4.5 | 2.3 | 5.8 | 3.2 | 5 | 12 | 5.9 | 0.9 | 8.2 | 3.5 | 4 | 21 | 4.7 | 2.7 | 4.7 | 4.7 | 7 |
| st | 18 | 4.4 | 1.5 | 4.0 | 4.8 | 5 | 14 | 5.5 | 0.4 | 5.2 | 5.8 | 4 | 20 | 5.1 | 2.1 | 4.4 | 5.9 | 7 |
| feed | 19 | 4.0 | 2.0 | 4.0 | 4.0 | 5 | 12 | 5.9 | 2.5 | 6.0 | 5.8 | 4 | 3 | 7.2 | 1.3 | 6.9 | 7.6 | 7 |
| illegal | 20 | 3.6 | 1.0 | 3.0 | 4.2 | 5 | 18 | 4.4 | 2.7 | 5.0 | 3.8 | 4 | 10 | 6.3 | 2.7 | 4.9 | 7.7 | 7 |
| water | 20 | 3.6 | 2.8 | 4.6 | 2.6 | 5 | 20 | 4.0 | 2.3 | 5.0 | 3.0 | 4 | 8 | 6.5 | 1.2 | 6.0 | 7.0 | 7 |
